# Supervised, but Not Home-Based, Isometric Training Improves Brachial and Central Blood Pressure in Medicated Hypertensive Patients: A Randomized Controlled Trial

**DOI:** 10.3389/fphys.2018.00961

**Published:** 2018-07-23

**Authors:** Breno Q. Farah, Sergio L. C. Rodrigues, Gustavo O. Silva, Rodrigo P. Pedrosa, Marilia A. Correia, Mauro V. G. Barros, Rafael Deminice, Poliana C. Marinello, Neil A. Smart, Lauro C. Vianna, Raphael M. Ritti-Dias

**Affiliations:** ^1^Graduate Program in Physical Education, University of Pernambuco, Recife, Brazil; ^2^Department of Physical Education, Rural Federal University of Pernambuco, Recife, Brazil; ^3^Sleep and Heart Laboratory, Pronto Socorro Cardiológico de Pernambuco, University of Pernambuco, Recife, Brazil; ^4^Graduate Program in Medicine, Universidade Nove de Julho, São Paulo, Brazil; ^5^Department of Physical Education, Faculty of Physical Education and Sport, State University of Londrina, Londrina, Brazil; ^6^School of Science and Technology, University of New England, Armidale, NSW, Australia; ^7^Faculty of Physical Education, Federal University of Brasilia, Brazilia, Brazil; ^8^Graduate Program in Rehabilitation Sciences, Universidade Nove de Julho, São Paulo, Brazil

**Keywords:** exercise, blood pressure, cardiovascular system, hypertension, resistance training

## Abstract

Meta-analyses have shown that supervised isometric handgrip training reduces blood pressure in hypertensives. However, the mechanism(s) underlying these effects in medicated hypertensive patients, as well as the effects from home-based exercise training, is uncertain. The purpose of this study was to compare the effects of supervised and home-based isometric handgrip training on cardiovascular parameters in medicated hypertensives. In this randomized controlled trial, 72 hypertensive individuals (38–79 years old, 70% female) were randomly assigned to three groups: home-based, supervised isometric handgrip training or control groups. Home-based and supervised isometric handgrip training was completed thrice weekly (4 × 2 min at 30% of maximal voluntary contraction, with 1-min rest between bouts, alternating the hands). Before and after 12 weeks brachial, central and ambulatory blood pressures (BP), arterial stiffness, heart rate variability, vascular function, oxidative stress and inflammation markers were obtained. No significant (*p* > 0.05) effect was observed for ambulatory BP, arterial stiffness, heart rate variability, vascular function and oxidative stress and inflammatory markers in all three groups. Brachial BP decreased in the supervised group (Systolic: 132 ± 4 vs. 120 ± 3 mmHg; Diastolic: 71 ± 2 vs. 66 ± 2 mmHg, *p* < 0.05), whereas no significant differences were observed in the home-based (Systolic: 130 ± 4 vs. 126 ± 3 mmHg; diastolic: 73 ± 3 vs. 71 ± 3 mmHg) and control groups (*p* > 0.05). Supervised handgrip exercise also reduced central BP systolic (120 ± 5 vs. 109 ± 5 mmHg), diastolic (73 ± 2 vs. 67 ± 2 mmHg); and mean BP (93 ± 3 vs. 84 ± 3 mmHg), whereas no significant effects were found in the home-based (Systolic: 119 ± 4 vs. 115 ± 3 mmHg; Diastolic: 74 ± 3 vs. 71 ± 3 mmHg) and control groups (*p* > 0.05). In conclusion, supervised, but not home-based, isometric training lowered brachial and central BP in hypertensives.

## Introduction

Hypertension is a disease characterized by sustained elevations in resting blood pressure (BP) and affects more than 1 billion people (Kearney et al., 2005). Elevated BP is directly related to cardiovascular or renal morbidity and mortality, accounting for 13% of total deaths worldwide (Lewington et al., 2002). Therefore, the principal goal of antihypertensive therapy is reducing resting BP to within target normal ranges (<130/80 mmHg), via lifestyle modification (including exercise training) in combination with pharmacotherapy (Whelton et al., 2018).

Meta-analyses have shown that isometric training, specially handgrip exercise, reducesbrachialsystolic and diastolic BPin hypertensive patients approximately 7 and 5 mmHg, respectively (Cornelissen and Smart, 2013; Carlson et al., 2014; Inder et al., 2016; Jin et al., 2017). These decreases seem to be greater than those observed after aerobic training. Isometric exercise was recently recommended as an adjunct treatment for hypertension by the American Heart Association and American College of Cardiology joint clincial practice guideline (Carey et al., 2018), however, most of the existing studies only analyzed brachial BP, limiting the understanding of the cardiovascular effects of isometric handgrip training (Farah et al., 2017a).

The mechanisms underlying the effects of isometric on blood pressure are controversial and inconclusive (Millar et al., 2014; Farah et al., 2017a). In practical terms, isometric exercise promotes repeated sympathetic responses through activation of neural reflexes associated with repeated episodes of local ischaemia followed by increased shear stress. Regarding the neural effects, studies have shown improvements in autonomic balance after handgrip isometric training (Taylor et al., 2003; Millar et al., 2013), which could be explained by an acute baroreflex enhancement after a period training (Teixeira et al., 2018). These local effects have been attributed toimprovements in local endothelial function (McGowan et al., 2006, 2007) and a reduction in oxidative stress (Peters et al., 2006). As endothelial function, cardiac autonomic modulation, and oxidative stress are directly related to inflammation and arterial stiffness in patients with hypertension (Kinlay et al., 2001; Casey et al., 2011; Sun, 2015; Herder et al., 2017), isometric handgrip training can also potentially moderate these clincial markers.

The protocol most frequently used in previous studies is supervised isometric handgrip training with 4 × 2 min at 30% of maximal voluntary contraction (MVC) with 1-min rest between bouts, alternating hands, thrice weekly. However, due to simplicity, isometric handgrip training can be useful as a home-based exercise, improving adherence, a primary concern in exercise program interventions. However, no study has previously analyzed the effects of home-based isometric handgrip training on cardiovascular parameters in hypertensive patients. Furthermore, the effects of isometric handgrip training in unmedicated individuals is well documented (Millar et al., 2014). In contrast, it remains unclear whether isometric handgrip training also improves blood pressure in patients taking anti-hypertensive medication.

This study aimedto compare the effects of supervised and unsupervised home-based isometric handgrip training on cardiovascular parameters in medicated hypertensive patients. Our hypothesis is that there is no difference between supervised or home-based isometric handgrip training.

## Materials and Methods

### Trial Design

This randomized controlled trial was registered with the www.clinicaltrials.gov database under the registration number NCT02348138 and is part of the ISOPRESS network (Farah et al., 2017b). The study procedures were approved by the Institutional Review Board in compliance with the Brazilian National Research Ethics System Guidelines. Written informed consent was obtained from each patient before participation.

### Participants

Medicated hypertensive patients were recruited through local media advertising and through flyers distributed in hospitals in the surrounding area of the University of Pernambuco, Brazil. Patients were included if they met the following criteria: (a) use of anti-hypertensive medications (b) >18 years old, (c) no diabetes, (d) no cardiovascular disease (other than hypertension), (e) did not present limitations to performance of isometric handgrip training, (f) were not involved in regular physical activity programs.

Patients were excluded from the study if they: (a) changed the type or dose antihypertensive medication, (b) joined an additional physical exercise program, (c) attended fewer than 80% of sessions in the home-based or supervised groups.

### Randomization and Allocation

The participants were block randomized using a random number table, stratified for sex and baseline brachial systolic BP (by a researcher not involved directly in the recruitment and data collection), into three groups: home-based isometric handgrip training, supervised isometric handgrip training and control group. Allocation was concealed to the researchers conducting measurements.

### Interventions

Patients assigned to the home-based and supervised isometric handgrip training group trained three times per week, for a total of 12 weeks. Each session consisted of four sets of 2-min isometric contractions (alternating the hands) performed using a handgrip dynamometer using (Zhongshan Camry Electronic Co., Ltd., Zhongshan Guangdong, China) at 30% of MVC and 1-min rest interval, load adjustments were performed in the sixth week (Farah et al., 2014).

The MVC was evaluated for both arms prior to the start of the study in all patients. The MVC test was conducted by requesting participants to undertake three measurements on each arm, with an interval of 1 min between each maximal effort. The result was the highest value of the three measurements. The intraclass correlation coefficient for the MVC test was 0.986 for the non-dominant arm and 0.989 for the dominant arm (Farah et al., 2014).

The only baseline difference between the two training groups was that the supervised group trained in the University laboratory, whereas the home-based group did their first session in the laboratory, to become familiarized with the technique and to adjust the load, thereafter the patients performed all sessions at home.

Patients assigned to the control group were advised to maintain dietary habits and physical activity levels. The isometric exercise program was offered to the control group after completion of the study for ethical reasons.

### Cardiovascular Parameters Measurements

Prior to all cardiovascular measurements, the patients were instructed to: (a) eat a light meal before arriving at the laboratory; (b) to avoid moderate-to-vigorous physical activity for at least 24 h prior to the visit, and; (c) avoid smoking, alcohol and caffeine ingestion for at least 12 h. In the laboratory, a rest period of 10 min in the supine position was required prior to the measurements. All measurements were taken in the supine position in a quiet environment, with a controlled temperature between 22 and 24°C. In addition, all data were collected by researchers blinded to the group allocations.

Cardiovascular measurements were obtained at baseline and after 12 weeks of intervention. Brachial, central, and ambulatory BP, arterial stiffness, heart rate variability, vascular function, oxidative stress and inflammation markers were obtained at the same time of day. The assessments followed the same order: heart rate variability, brachial and central BP, arterial stiffness, and vascular function. Ambulatory BP and blood sample collection were taken on another day. The post intervention assessments were obtained at least after 72 h of the last exercise session.

#### Brachial BP

Brachial BP measurements were performed using Omron HEM equipment 742. After remaining in a supine position for 10 min, at least three consecutive measurements within an interval of 4 mmHg were performed with 1-min interval between measurements. Measurements were performed on the right arm and with the proper cuff size for arm circumference. The value used was the average of the last two measures (Sociedade Brasileira de Cardiologia et al., 2010). Intraclass correlation coefficient for systolic BP was 0.85 and diastolic BP 0.92 (Gerage et al., 2015).

#### Central BP

Central systolic and diastolic BP were obtained by pulse wave analysis, recorded in the left radial artery using applanation tonometry (SphygmoCor, AtCor Medical, Australia) and the validated transfer function algorithm provided by the Sphygmocor^®^ software obtained the central values of systolic diastolic and mean BP (equivalent to the pressure wave measured by an invasive catheter (Siebenhofer et al., 1999). To enhance the accuracy of measurements, only those values whose quality index exceeded 90% were utilized.

#### Ambulatory BP

The ambulatory BP readings over a 24-h period were obtained using a oscillometric device (Dyna-MAPA, Cardios, Brazil) programmed to take measurements every 15 min during the daytime and 30 min at night time, using the procedures previously described (O’Brien et al., 2013; Rodrigues et al., 2014). Additionally, patients were advised to report major routine activities, such as: meals, displacements, medications, bedtime, and wake-up. These reports were taken into account during the ambulatory BP data analysis.

#### Arterial Stiffness Markers

Pulse wave velocity was measured using high-fidelity applanation tonometry (Sphygmocor, ATCOR Medical, Australia) following the guidelines of the Clinical Application of Arterial Stiffness, Task Force III (Van Bortel et al., 2002). For the central pulse wave velocity (cPWV), the distance between the suprasternal notch and carotid artery and the distance between the suprasternal notch and the femoral artery were measured using a standard tape. The distance between the two arteries was divided by the time difference in both markers. For the measurement of the peripheral pulse wave velocity (pPWV), the distance between the suprasternal notch and the femoral artery, and the suprasternal notch and the dorsalis pedis artery were also measured using a standard tape. The distance between the two arteries was divided by the time difference in both markers. Simultaneously an electrocardiogram was assessed to obtain heart rate and, according to a “foot-to-foot” method, the time difference between the points was measured.

#### Heart Rate Variability

Heart rate variability was assessed from the RR intervals obtained by a heart rate monitor (RS800CX, Polar Electro, Finland). Patients remained in the supine position for 10 min and we analyzed at least 5 min of stationary R-R interval data. All analyses were performed with Kubios HRV software (Biosignal Analysis and Medical Imaging Group, Joensuu, Finland) by a single evaluator blinded to the group allocations. Intraclass correlation coefficient for this evaluator ranged from 0.990 to 0.993 (Farah et al., 2016), using the recommendations of the Task Force for heart rate variability (Malik et al., 1996).

The following time-domain variables were examined for each recording: standard deviation of all RR intervals (SDNN), root mean square of the squared differences between adjacent normal RR intervals (RMSSD), and the percentage of adjacent intervals over 50 ms (PNN50). Additionally, frequency-domain variables were calculated via an autoregressive method. The signals operating at frequencies between 0.04 and 0.4 Hz were considered physiologically significant with the low frequency component represented by oscillations between 0.04 and 0.15 Hz, and high frequency component represented by oscillations between 0.15 and 0.4 Hz. The power of each spectral component was normalized by dividing the power of each spectrum band by the total variance, minus the value of the very low frequency band (<0.04 Hz) and multiplying the result by 100. To interpret the results, the low frequency and high frequency normalized components were considered, respectively, as representative of predominantly combined parasympathetic-sympathetic and parasympathetic modulation of the heart, and the ratio between these bands (LF/HF) was defined as the cardiac sympathovagal balance.

#### Vascular Function

Brachial artery diameter and blood flow velocity were measured using a high-resolution duplex-Doppler ultrasound (Apogee 3500, SIUI, China) following the relevant guideline (Thijssen et al., 2011). Resting brachial artery diameterand blood velocity waveforms were continuously recorded over 120 s. Recordings of all vascular variables were analyzed offline using specialized edge-detection software (Cardiovascular Suite, Quipu, Italy).

#### Oxidative Stress and Inflammation Markers

Blood samples (4 ml) were collected in tubes containing EDTA in the hospital, homogenized by inversion, and then were centrifuged at 1500 rpm for 15 min. Thereafter, the plasma were separated, placed in Eppendorf tubes, and kept at -80°C until analysis. Oxidative stress was assessed on plasma samples by the quantification of the advanced oxidized protein products (AOPP), which reflects protein oxidation of inflammatory nature and malondialdehyde (MDA), a final product of the lipoperoxidation reaction. Total thiol levels, were measured to estimate non-enzymatic antioxidant defenses. AOPP (Witko-Sarsat et al., 1996) and MDA (Spirlandeli et al., 2014) levels were assessed by the methods previously described and plasmatic total thiols were quantified by the protocol described by Costa et al. (2006). Evaluation of interleukin-1β (IL-1β), interleukin-10 (IL-10), interleukin-6 (IL-6), tumor necrosis factor-α (TNF-α), C-reactive protein (CRP) were performed in plasma samples using available commercial kits, following the manufacturer’s instructions (Invitrogen, Carlsbad, CA, United States). For the CRP analysis, plasma samples were diluted 4000 times, for the other analysis, the samples were used without previous dilution. All the assays were determined in duplicates. The coefficient of variation for all measurements was less than 8% (IL-1B 1.7%, IL-10 2.2%, IL-6 1.1%, TNF-a 7.8%, PCR 5.2%, AOPP 3.4%, MDA 2.1%, Total Thiols 1.5%).

### Statistical Analysis

The data were stored and analyzed using the Statistical Package for the Social Sciences (SPSS Version 17.0 for Windows). Normality was checked using the Shapiro-Wilk test and the Levene test was used to analyze the homogeneity of variances. Continuous variables were summarized as mean, standard deviations or 95% confidence interval, whereas categorical variables were summarized as relative frequencies. The clinical characteristics among groups compared using one-way analysis of variance for continuous variables and chi-square test for categorical variables.

To compare the effects of interventions on cardiovascular risk indicators, Generalized Estimating Equations (GEE) were used, followed by a *post hoc* pairwise comparison using the Bonferroni correction for multiple comparisons. Effect size (ES) was used to estimate the magnitude of differences within the same group. The significance level was set at *P* < 0.05 (two-tailed testing) for all analyses.

## Results

**FIGURE 1:**
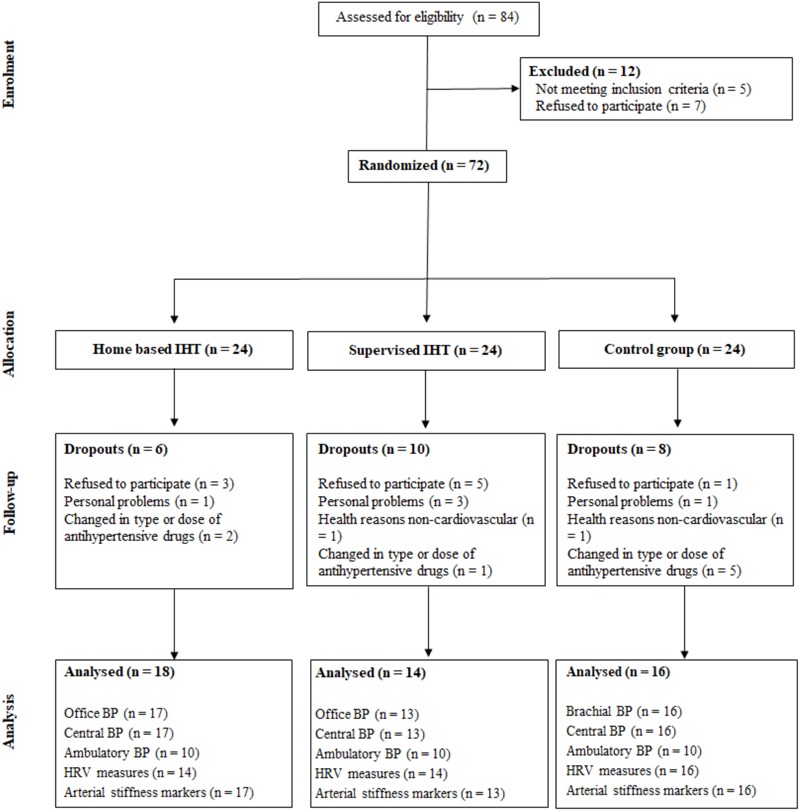
Flowchart of study. IHT, isometric handgrip training. BP, blood pressure. HRV, heart rate variability.

Recruitment and intervention periods occurred between July 2015 and August 2016. The study flowchart is shown in **Figure 1**. Some subjects reported pain in the hand joints during exercise, but this was not sufficient to leave the study. One woman from the home-based group reported dyspnea during exercise in the second week and tachycardia in the sixth and eighth weeks, however, clinical examinations were performed and no abnormalities were observed. All baseline characteristics were similar among the groups (**Table 1**). Seventy percent of the patients were women, age range 38–79 years old (95% confidence interval 56.7–62.4 years old).

**Table 1:** General characteristics of experimental groups at baseline.

Variables	Control *n* = 16	Home-based *n* = 18	Supervise *n* = 14	*p*
Age (years)	58 ± 3	61 ± 2	59 ± 2	0.753
Weight (kg)	83.4 ± 3.9	80.9 ± 4.0	79.1 ± 5.8	0.827
Height (m)	1.61 ± 0.02	1.61 ± 0.02	1.62 ± 0.03	0.970
Body mass index (kg/m^2^)	31.7 ± 1.3	31.1 ± 1.2	29.9 ± 1.7	0.655
Brachial systolic BP (mmHg)	132 ± 4	130 ± 4	129 ± 5	0.908
Brachial diastolic BP (mmHg)	71 ± 2	73 ± 3	73 ± 2	0.825
Maximal voluntary contraction (kgf)	29.9 ± 2.3	31.7 ± 2.1	29.7 ± 3.1	0.818
Education level (% low)	40	65	79	0.312
Sex (% women)	69	72	69	0.933
Calcium channel blocker (%)	23	17	19	0.904
Diuretic (%)	77	44	38	0.083
β-blocker (%)	23	22	20	0.953
ACE inhibitor (%)	15	33	6	0.124
Angiotensin receptor blockers (%)	61	77	81	0.386

*Values are presented as mean ± standard error or frequency. BP, blood pressure; ACE, angiotensin converting enzyme.*

**FIGURE 2:**
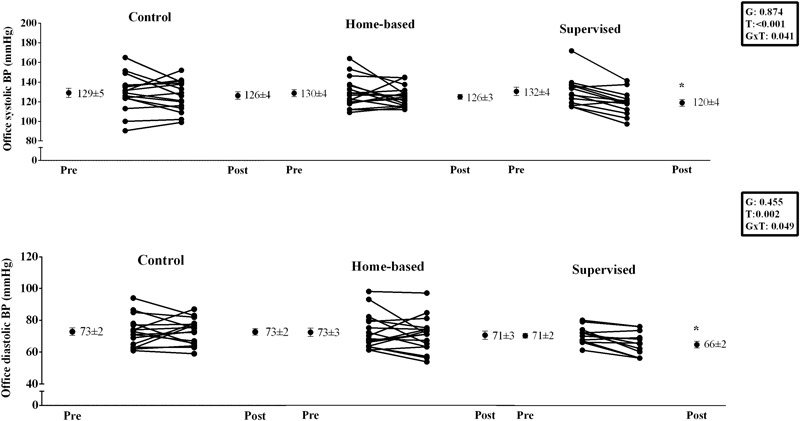
Effects of home-based and supervised isometric handgrip training onbrachial blood pressure. ^∗^ significant difference from Pre.

**FIGURE 3:**
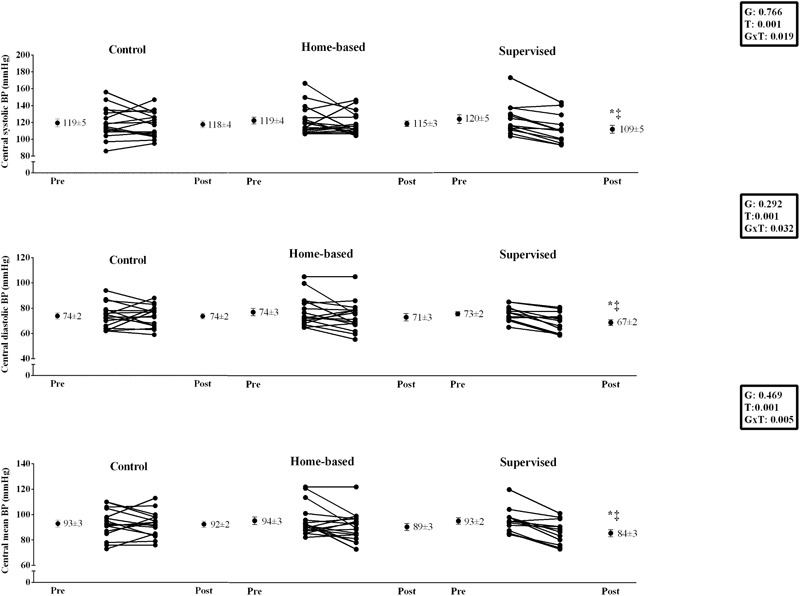
Effects of home-based and supervised isometric handgrip training on central blood pressure. ^∗^significant difference from Pre; ^‡^significant difference from control group.

Supervised handgrip training produced a significant (*P* < 0.05) decrease in brachial systolic (132 ± 4 vs. 120 ± 3 mmHg, ES = 0.94) and diastolic BP (71 ± 2 vs. 66 ± 2 mmHg, ES = 0.69), which was not observed for home-based and control groups (Systolic BP – Home-based: 130 ± 4 vs. 126 ± 3 mmHg, ES = 0.24; Control: 129 ± 5 vs. 126 ± 4 mmHg, ES = 0.17; Diastolic BP – Home-based: 73 ± 3 vs. 71 ± 3 mmHg, ES = 0.16; Control: 73 ± 2 vs. 73 ± 2 mmHg, ES = 0.0) (see **Figure 2**). Supervised handgrip training caused a significant reduction in central BP (Systolic: 120 ± 5 to 109 ± 4 mmHg, ES = 0.61;Diastolic: 73 ± 2 vs. 67 ± 2 mmHg, ES = 0.83; mean BP: 93 ± 2 to 84 ± 3 mmHg, ES = 0.83). Home-based handgrip training reduced mean BP (94 ± 3 vs. 89 ± 3 mmHg, ES = 0.40), whereas no difference was found for central systolic (119 ± 4 vs. 115 ± 3 mmHg, ES = 0.27) and diastolic BP (74 ± 3 vs. 71 ± 3 mmHg, ES = 0.24) (**Figure 3**). A significant positive correlation was observed between change in brachial and central BP in the supervised group (systolic BP: *r* = 0.779, *p* = 0.002; diastolic BP: *r* = 0.919, *p* < 0.001). Patients in the home-based group who responded to training had higher baseline systolic BP compared to non-responders (139 ± 5 vs. 120 ± 3 mmHg, *p* = 0.005).

No significant pre-post intervention differences were observed among the groups for ambulatory BP, arterial stiffness, heart rate variability parameters, vascular function, oxidative stress and inflammatory markers (*P* > 0.05) (**Tables 2, 3**). Results of additional analyses in hypertensive participants who did not take beta blocker medication are presented in the Supplementary Document 1.

**Table 2:** Effects of home-based and supervised isometric handgrip training and control on ambulatory blood pressure in hypertensives.

Variables	Supervised		Home-based		Control		*P*
	Pre	Post	Pre	Post	Pre	Post	
**24 h**							
Systolic blood pressure (mmHg)	121 ± 4	122 ± 3	117 ± 2	117 ± 2	115 ± 4	115 ± 5	0.650
Mean blood pressure (mmHg)	98 ± 4	98 ± 2	93 ± 2	93 ± 2	90 ± 2	90 ± 3	0.463
Diastolic blood pressure (mmHg)	79 ± 4	79 ± 3	72 ± 2	73 ± 3	70 ± 2	70 ± 3	0.340
**Awake period**							
Systolic blood pressure (mmHg)	122 ± 4	123 ± 3	117 ± 2	121 ± 2	117 ± 4	117 ± 5	0.627
Mean blood pressure (mmHg)	100 ± 4	99 ± 2	93 ± 2	96 ± 2	92 ± 2	93 ± 4	0.611
Diastolic blood pressure (mmHg)	80 ± 4	79 ± 3	73 ± 3	75 ± 2	71 ± 2	71 ± 3	0.626
**Asleep period**							
Systolic blood pressure (mmHg)	118 ± 5	115 ± 4	115 ± 3	116 ± 4	106 ± 4	109 ± 4	0.973
Mean blood pressure (mmHg)	96 ± 5	93 ± 4	91 ± 2	92 ± 4	83 ± 3	85 ± 3	0.971
Diastolic blood pressure (mmHg)	76 ± 3	74 ± 4	70 ± 3	72 ± 4	63 ± 3	64 ± 3	0.843

**Table 3:** Effects of home-based and supervised isometric handgrip training and control on cardiovascular parameters in hypertensives.

Variables	Supervised		Home-based		Control		*P*
	Pre	Post	Pre	Post	Pre	Post	
**Arterial stiffness parameters**							
Pulse pressure (mmHg)	13.9 ± 3.3	11.6 ± 2.6	13.2 ± 2.1	13.2 ± 2.0	13.5 ± 2.2	13.3 ± 1.8	0.620
Augmentation index (%)	26.6 ± 4.0	25.4 ± 4.0	27.2 ± 2.2	27.2 ± 2.7	27.9 ± 3.2	29.1 ± 2.4	0.841
Central PWV (m/s)	8.4 ± 0.4	7.7 ± 0.3	9.8 ± 0.5	8.8 ± 0.3	8.7 ± 0.5	8.8 ± 0.5	0.219
Peripheral PWV (m/s)	8.3 ± 0.3	8.5 ± 0.3	8.9 ± 0.4	8.9 ± 0.5	9.3 ± 0.4	9.4 ± 0.4	0.960
**Heart rate variability**							
SDNN (ms)	39 ± 4	38 ± 4	29 ± 4	31 ± 4	38 ± 6	39 ± 3	0.924
RMSSD (ms)	31 ± 4	32 ± 5	21 ± 4	27 ± 4	33 ± 8	30 ± 4	0.470
pNN50 (%)	12 ± 4	13 ± 5	5 ± 3	9 ± 4	10 ± 4	10 ± 3	0.687
Low frequency (nu)	53 ± 6	57 ± 7	54 ± 5	47 ± 5	52 ± 3	52 ± 3	0.302
High frequency (nu)	47 ± 6	43 ± 7	46 ± 5	53 ± 5	48 ± 3	48 ± 3	0.302
LF/HF	2.00 ± 0.74	2.26 ± 0.54	1.88 ± 0.50	1.16 ± 0.23	1.28 ± 0.21	1.42 ± 0.24	0.215
**Vascular function**							
Resting brachial diameter (cm)	4.01 ± 0.21	4.11 ± 0.22	4.17 ± 0.21	3.97 ± 0.27	3.83 ± 0.20	3.99 ± 0.20	0.122
Resting shear rate (s)	122 ± 10	105 ± 14	111 ± 15	110 ± 10	111 ± 15	96 ± 12	0.694
**Oxidative stress and inflammation markers**							
AOPP (μM)	88 ± 21	22 ± 6^∗^	71 ± 11	57 ± 25	67 ± 25	11 ± 3^∗^	0.032
MDA (μM)	1.54 ± 0.22	1.15 ± 0.14	1.52 ± 0.14	1.12 ± 0.13	1.53 ± 0.18	1.20 ± 0.06	0.906
Total Thiols (μM)	264 ± 19	234 ± 32	260 ± 31	326 ± 95	281 ± 13	310 ± 76	0.453
IL-6 (pg/mL)	1.36 ± 0.09	1.42 ± 0.19	1.24 ± 0.15	1.49 ± 0.16	1.36 ± 0.25	1.57 ± 0.15	0.669
IL-10 (pg/mL)	4.6 ± 0.5	4.0 ± 0.6	5.2 ± 0.4	4.9 ± 0.4	4.7 ± 0.6	4.4 ± 0.5	0.952
IL-1b (pg/mL)	0.32 ± 0.02	0.30 ± 0.02	0.37 ± 0.02	0.34 ± 0.03	0.37 ± 0.02	0.43 ± 0.04	0.328
CRP (pg/mL)	899 ± 201	977 ± 269	1812 ± 279	1446 ± 485	1126 ± 226	1188 ± 322	0.592
TNF-alpha (pg/mL)	3.2 ± 0.2	4.9 ± 0.7	3.5 ± 0.2	3.9 ± 0.6	9.6 ± 3.8	6.6 ± 1.1	0.141

*^∗^Significant difference from pre.*

## Discussion

The main finding of this study was that 12 weeks of supervised isometric handgrip training significantly reduced brachial BP, and demonstrated for the first time that supervised isometric handgrip training also decreased central systolic and mean BP in hypertensives. In contrast, home-based isometric handgrip training did not improve brachial, central, ambulatory BP, arterial stiffness, heart rate variability, vascular function, oxidative stress and inflammatory markers in hypertensive subjects.

The beneficial effects of supervised isometric handgrip training on brachial BP observed in this study correspond with previous findings (Cornelissen and Smart, 2013; Carlson et al., 2014; Inder et al., 2016; Jin et al., 2017). Our study demonstrated a net-effect reduction of approximately 9 and 6 mmHg for brachial systolic and diastolic BP, respectively, corresponding to a moderate-high ES (0.94 and 0.69). A recent meta-analysis demonstrated reductions of 8 mmHg for systolic BP and 4 mmHg for diastolic BP (Jin et al., 2017), which are similar to the results of our present study. The analysis of individual responses also confirmed the beneficial effects of supervised isometric handgrip training on brachial BP. In fact, 85% of patients reduced systolic BP by more than 5 mmHg and 77% of patients reduced diastolic BP by more than 2 mmHg.

In addition to the improvements in brachial BP, we demonstrated for the first time that supervised isometric training reduced central BP, which is considered a better discriminant of cardiovascular risk and target organ damage than brachial BP (Wang et al., 2009). The observed reductions of 6 and 4 mmHg for central systolic and diastolic BP, respectively, are estimated to reduce the risk of cardiovascular mortality by approximately 25% (Wang et al., 2009). Individual analyzes demonstrated reduced central BP, in approximately 90% of patients, in the supervised isometric handgrip training group, demonstrating efficacy of this intervention.

Ambulatory BP did not change after isometric handgrip training. Similarly, Stiller-Moldovan et al. (2012) did not observe reduced ambulatory BP after 8 weeks of supervised isometric handgrip training in medicated hypertensive participants. Recently, Pagonas et al. (2017) also did not observe reductions in ambulatory BP after supervised in hypertensive individuals. Previous studies have shown that ambulatory BP is a better marker of cardiovascular risk than office BPs (brachial or central BP) (Huang et al., 2011). Therefore, these results suggest that the isometric handgrip training may not be effective in reducing the cardiovascular risk of hypertensive patients. However, in the present study there was 40% attrition for the ambulatory BP analysis, which may have influenced statistical power (Supplementary Document 2).

In contrast to our hypothesis, the results of the present study indicated no effects of home-based isometric handgrip training on BP, suggesting that patients did not correctly perform or did not perform the isometric handgrip training. The absence of a long familiarization period to isometric exercise (as patients only performed one familiarization session), the raised perception of forearm discomfort in the last seconds of exercise (Brown and Bray, 2015), and the low educational level of the patients may explain our results.

Cardiac autonomic modulation did not change after 12-weeks isometric handgrip training. Stiller-Moldovan et al. (2012) also did not observe an improvement after 8 weeks of supervised isometric handgrip training in medicated hypertensive participants. These results remained even after exclusion of patients taking beta-blockers. In contrast, Taylor et al. (2003) noted marked improvements in high frequency spectral power in uncontrolled hypertensive after supervised isometric handgrip training. These results taken together may suggest that isometric handgrip training could present some improvement in cardiovascular parameters in patients with poorly controlled BP levels. However, future studies should consider focusing on target populations of hypertensive subjects with BP above the normal range.

Increased oxidative stress and inflammation are thought to play a key role in the pathophysiology of hypertension (Vaziri and Rodriguez-Iturbe, 2006). Improvements in oxidative stress were proposed as a potential mechanism to improve BP after isometric handgrip training in a single group small study (Peters et al., 2006). Interestingly, supervised handgrip exercise appears to improve central BP independently of oxidative stress and inflammation. Our data demonstrated no significant change in oxidative stress (AOPP, MDA, total thiols) or inflammatory mediators (IL-1β, IL-10, IL-6, TNF-α, and CRP) after 12 weeks. These responses may be expected as cardiac autonomic function was unchanged (Johnston and Webster, 2009), as was body composition. Since isometric handgrip exercise does not promote fat reduction and increased lean mass, it is therefore unsurprising that there was no improvement in the inflammatory and oxidative stress profiles (Wu et al., 2013).

Arterial stiffness did not change after the intervention, suggesting exercise does not alter the vessel structure in the short term. These responses coupled with no change in the cardiac autonomic modulation, oxidative stress and cytokines, suggest that other mechanisms may act to reduce BP after isometric exercise. A recent study observed that isometric handgrip acutely increases spontaneous cardiac baroreflex sensitivity (Teixeira et al., 2018). Given that acute exercise responses have been related to chronic training effects, it is possible that baroreflex sensitivity may explain the anti-hypertensive effects of isometric exercise (Somani et al., 2017). Thus, future studies should analyze the chronic effects of isometric exercise on baroreflex sensitivity.

Isometric handgrip training has emerged as a potential new tool to improve BP in the hypertensive population (Cornelissen and Smart, 2013; Carlson et al., 2014; Inder et al., 2016; Jin et al., 2017; Carey et al., 2018) Advantages of this exercise modality include simplicity and a reduced time commitment to perform the protocol, which could be performed at home. However, the results of the current study failed to show efficacy of using of this exercise modality at home. Future studies should also address whether other devices or monitoring strategies can improve the effectiveness of home-based isometric handgrip training.

A major limitation of our study was that the isometric handgrip equipment used for training does not log are cord of training sessions, thus, it is not possible to affirm that participants adhered to the intervention. The sample size did not allow for stratified analysis by medication, which would enable an understanding of the mechanism(s) of blood pressure lowering after isometric handgrip training. There was standardization of the time of day that the intervention was performed, which may have generated different cardiovascular responses (de Brito et al., 2015). Baroreflex sensitivity was not measured and the understanding of the mechanisms of blood pressure lowering after isometric handgrip training was limited. Finally, generalizability of results to other populations (advanced hypertension or clinical other populations) should be performed with caution.

In conclusion, 12-weeks supervised isometric handgrip training reduced brachial and central BP, although these variables were unchanged in the home-based handgrip training group. In addition, neither supervised or home-based training improved ambulatory BP, arterial stiffness, heart rate variability, vascular function, oxidative stress and inflammatory mediators.

## Author Contributions

BF, SR, LV, RR-D, and MB were responsible for the conception and design of the study. BF, SR, GS, and MC worked on data collection. MC, RD, and PM worked on data analysis. All authors read contributed to the writing and approved the final version of the manuscript.

## Conflict of Interest Statement

The authors declare that the research was conducted in the absence of any commercial or financial relationships that could be construed as a potential conflict of interest. The reviewer WS and handling editor declared their shared affiliations.
