# Supplementary material for: Supervised, but Not Home-Based, Isometric Training Improves Brachial and Central Blood Pressure in Medicated Hypertensive Patients: A Randomized Controlled Trial
Source: Front Physiol. 2018 Jul 23;9:961. doi: 10.3389/fphys.2018.00961 (PMC6065303; doi:10.3389/fphys.2018.00961)
Supplement: Supplementary file 1 [file Table_1.docx]

Supplementary document 1. Effects of home-based and supervised isometric handgrip training on brachial, central and ambulatory blood pressure, heart rate variability in hypertensive who did not taken the beta blocker medication.

| **Variables** | **Supervised (n =10)** | | | | **Home-based (n =13)** | | | **Control (n =13)** | | | ***P*** |
| --- | --- | --- | --- | --- | --- | --- | --- | --- | --- | --- | --- |
|  | **Pre** | **Post** | **ES** | **Pre** | | **Post** | **ES** | **Pre** | **Post** | **ES** |  |
| ***Brachial blood pressure*** |  |  |  |  | |  |  |  |  |  |  |
| Systolic blood pressure (mmHg) | 132 ± 6 | 118 ± 4* | 0.92 | 129 ± 4 | | 126 ± 3 | 0.24 | 131 ± 5 | 125 ± 4 | 0.38 | 0.036 |
| Diastolic blood pressure (mmHg) | 71 ±2 | 65 ± 2 | 1.00 | 74 ± 3 | | 72 ± 3 | 0.19 | 74 ± 3 | 73 ± 2 | 0.09 | 0.088 |
| ***Central blood pressure*** |  |  |  |  | |  |  |  |  |  |  |
| Systolic blood pressure (mmHg) | 119 ± 6 | 106 ± 5* | 0.78 | 118 ± 3 | | 116 ± 4 | 0.16 | 121 ± 5 | 117 ± 4 | 0.26 | 0.018 |
| Diastolic blood pressure (mmHg) | 73 ± 2 | 66 ± 3 | 0.92 | 75 ± 3 | | 71 ± 4 | 0.33 | 75 ± 3 | 74 ± 2 | 0.11 | 0.052 |
| Mean blood pressure (mmHg) | 93 ± 3 | 83 ± 3*‡ | 1.11 | 94 ± 3 | | 89 ± 3 | 0.48 | 94 ± 3 | 92 ± 2 | 0.23 | 0.003 |
| ***24 hours*** |  |  |  |  | |  |  |  |  |  |  |
| Systolic blood pressure (mmHg) | 115 ± 4 | 115 ± 7 | 0.00 | 116 ± 2 | | 116 ± 2 | 0.00 | 117 ± 4 | 119 ± 2 | 0.18 | 0,931 |
| Mean blood pressure (mmHg) | 91 ± 3 | 90 ± 5 | 0.08 | 93 ± 2 | | 92 ± 3 | 0.11 | 94 ± 4 | 96 ± 2 | 0.18 | 0,739 |
| Diastolic blood pressure (mmHg) | 71 ± 2 | 70 ± 4 | 0.11 | 73 ± 2 | | 72 ± 3 | 0.11 | 74 ± 4 | 72 ± 3 | 0.08 | 0,521 |
| ***Awake period*** |  |  |  |  | |  |  |  |  |  |  |
| Systolic blood pressure (mmHg) | 117 ± 4 | 116 ± 8 | 0.05 | 116 ± 2 | | 120 ± 2 | 0.58 | 119 ± 4 | 121 ± 3 | 0.16 | 0,830 |
| Mean blood pressure (mmHg) | 93 ± 3 | 92 ± 6 | 0.07 | 94 ± 2 | | 96 ± 2 | 0.29 | 96 ± 4 | 97 ± 2 | 0.09 | 0,797 |
| Diastolic blood pressure (mmHg) | 73 ± 2 | 72 ± 4 | 0.11 | 75 ± 2 | | 75 ± 3 | 0.00 | 76 ± 4 | 78 ± 2 | 0.18 | 0,689 |
| ***Asleep period*** |  |  |  |  | |  |  |  |  |  |  |
| Systolic blood pressure (mmHg) | 107 ± 4 | 108 ± 5 | 0.07 | 114 ± 2 | | 114 ± 4 | 0.00 | 113 ± 4 | 112 ± 3 | 0.08 | 0,976 |
| Mean blood pressure (mmHg) | 84 ± 3 | 84 ± 5 | 0.00 | 90 ± 2 | | 91 ± 4 | 0.09 | 90 ± 4 | 90 ± 3 | 0.00 | 0,997 |
| Diastolic blood pressure (mmHg) | 64 ± 3 | 65 ± 4 | 0.09 | 70 ± 2 | | 71 ± 4 | 0.09 | 71 ± 4 | 72 ± 3 | 0.08 | 0,985 |
| ***Heart Rate Variability*** |  |  |  |  | |  |  |  |  |  |  |
| SDNN (ms) | 35 ± 3 | 37 ± 5 | 0.16 | 26 ± 3 | | 32 ± 4 | 0.49 | 39 ± 7 | 38 ± 4 | 0.05 | 0.574 |
| RMSSD (ms) | 25 ± 4 | 27 ± 5 | 0.15 | 18 ± 2 | | 28 ± 5 | 0.82 | 33 ± 9 | 30 ± 5 | 0.12 | 0.278 |
| pNN50 (%) | 8 ± 4 | 11 ± 4 | 0.25 | 3 ± 2 | | 11 ± 5 | 0.61 | 9 ± 4 | 10 ± 4 | 0.07 | 0.524 |
| Low frequency (nu) | 60 ± 6 | 62 ± 8 | 0.09 | 52 ± 5 | | 49 ± 6 | 0.16 | 52 ± 4 | 51 ± 5 | 0.06 | 0.777 |
| High frequency (nu) | 40 ± 6 | 38 ± 8 | 0.09 | 48 ± 5 | | 51 ± 6 | 0.16 | 48 ± 4 | 49 ± 5 | 0.06 | 0.777 |
| LF/HF | 2.47 ± 0.91 | 2.75 ± 0.62 | 0.12 | 1.82 ± 0.59 | | 1.25 ± 0.25 | 0.36 | 1.33 ± 0.26 | 1.39 ± 0.28 | 0.06 | 0.530 |

* signiﬁcant difference from Pre; ‡ signiﬁcant difference from control group. ES – effect size
