# Supplementary material for: Supervised, but Not Home-Based, Isometric Training Improves Brachial and Central Blood Pressure in Medicated Hypertensive Patients: A Randomized Controlled Trial
Source: Front Physiol. 2018 Jul 23;9:961. doi: 10.3389/fphys.2018.00961 (PMC6065303; doi:10.3389/fphys.2018.00961)
Supplement: Supplementary file 2 [file Table_2.docx]

Supplementary document 2. Effects of home-based and supervised isometric handgrip training on brachial blood pressure only patients with ambulatory blood pressure data.

| **Variables** | **Supervised (n =9)** | | | | **Home-based (n =10)** | | | **Control (n =10)** | | | ***P*** |
| --- | --- | --- | --- | --- | --- | --- | --- | --- | --- | --- | --- |
|  | **Pre** | **Post** | **ES** | **Pre** | | **Post** | **ES** | **Pre** | **Post** | **ES** |  |
| ***Brachial blood pressure*** |  |  |  |  | |  |  |  |  |  |  |
| Systolic blood pressure (mmHg) | 128 ± 3 | 120 ± 3 | 0.91 | 132 ± 4 | | 125 ± 3 | 0.78 | 130 ± 5 | 132 ± 4 | 0.15 | 0.228 |
| Diastolic blood pressure (mmHg) | 71 ±3 | 65 ± 3*‡ | 0.71 | 75 ± 3 | | 69 ± 3 | 0.67 | 73 ± 3 | 75 ± 2 | 0.26 | 0.037 |

* signiﬁcant difference from Pre; ‡ signiﬁcant difference from control group. ES – effect size
